# Supplementary material for: Changes in tooth brushing frequency and its associated factors from 2006 to 2014 among French adolescents: Results from three repeated cross sectional HBSC studies
Source: PLoS One. 2021 Mar 29;16(3):e0249129. doi: 10.1371/journal.pone.0249129 (PMC8007017; doi:10.1371/journal.pone.0249129)
Supplement: S1 Questionnaire — (PDF) [file pone.0249129.s002.pdf]

Questionnaire

Le questionnaire est présenté ici dans sa version complète destinée aux élèves les plus âgés. Les élèves de CM2, 6e et 5e avaient une version sans les questions 13, 17-19 (substances illicites) et 39-46 (vie sexuelle).

---

1 **Es-tu une fille ou un garçon ?**

1 ☐ Garçon
2 ☐ Fille

---

2 **En quelle classe es-tu ?**

4 ☐ Quatrième
5 ☐ Troisième
6 ☐ Seconde
7 ☐ Autre, précise :

---

3 **En quel mois es-tu né(e) ?**

1 ☐ Janvier (01)
2 ☐ Février (02)
3 ☐ Mars (03)
4 ☐ Avril (04)
5 ☐ Mai (05)
6 ☐ Juin (06)
7 ☐ Juillet (07)
8 ☐ Août (08)
9 ☐ Septembre (09)
10 ☐ Octobre (10)
11 ☐ Novembre (11)
12 ☐ Décembre (12)

4 **En quelle année es-tu né(e) ?**

☐	☐	☐	☐	☐	☐
1992	1993	1994	1995	1996	1997

5 **D'habitude, combien de fois par semaine prends-tu un petit déjeuner (plus qu'un bol de café, de thé, de lait ou qu'un verre de jus de fruit) ?**
◆ *Coche une case pour les 5 jours de la semaine et une autre pour le week-end.*

SEMAINE (5 jours de classe, du lundi au vendredi) :
1 ☐ Je ne prends jamais de petit déjeuner en semaine
2 ☐ Un jour
3 ☐ Deux jours
4 ☐ Trois jours
5 ☐ Quatre jours
6 ☐ Cinq jours

WEEK-END :
1 ☐ Je ne prends jamais de petit déjeuner le week-end
2 ☐ D'habitude, je ne prends un petit déjeuner qu'un seul matin du week-end (le samedi OU le dimanche)
3 ☐ D'habitude, je prends un petit déjeuner les deux matins du week-end (le samedi ET le dimanche)

6 **Combien de fois par semaine manges-tu ou bois-tu les aliments suivants ?**
◆ *Coche une case pour chaque ligne.*

	Jamais	Moins d'une fois par semaine	Une fois par semaine	2-4 jours par semaine	5-6 jours par semaine	Une fois par jour, tous les jours	Plusieurs fois par jour, tous les jours
1 Fruits	☐	☐	☐	☐	☐	☐	☐
2 Légumes	☐	☐	☐	☐	☐	☐	☐
3 Sucreries (bonbons, chocolat)	☐	☐	☐	☐	☐	☐	☐
4 Coca, soda ou autre boisson contenant du sucre	☐	☐	☐	☐	☐	☐	☐
5 Boissons « énergisantes » (Red-Bull, Dark Dog, Burn…)	☐	☐	☐	☐	☐	☐	☐
6 Boissons « énergisantes » associées à de l'alcool	☐	☐	☐	☐	☐	☐	☐

7 **Certains jeunes vont à l'école ou au lit en ayant faim parce qu'il n'y a pas assez de nourriture à la maison. Tous les combien cela t'arrive-t-il ?**
1 ☐ Toujours
2 ☐ Souvent
3 ☐ Parfois
4 ☐ Jamais

8 **Tous les combien te brosses-tu les dents ?**
1 ☐ Plus d'une fois par jour
2 ☐ Une fois par jour
3 ☐ Au moins une fois par semaine mais pas chaque jour
4 ☐ Moins d'une fois par semaine
5 ☐ Jamais

9 **Pour le moment, fais-tu un régime ou autre chose pour perdre du poids ?**
1 ☐ Non, mon poids est bon
2 ☐ Non, mais j'ai besoin de perdre du poids
3 ☐ Non, parce que j'ai besoin de grossir
4 ☐ Oui

Ton activité physique

Une activité physique est n'importe quelle activité qui augmente la vitesse des battements du cœur et fait se sentir essoufflé(e) par moments.
On peut pratiquer une activité physique en faisant du sport, en jouant avec des amis à l'école ou ailleurs, ou bien en allant à l'école en marchant.
Quelques exemples d'activité physique : courir, marcher vite, faire du roller, faire du vélo, danser, faire du skate, nager, faire du foot, du rugby, du basket ou du surf.
Pour la question suivante, additionne tout le temps que tu as passé à faire une activité physique au cours de chaque jour.

10 **Pendant les 7 derniers jours, combien de jours as-tu pratiqué une activité physique pour un total de 60 minutes (1 heure) au moins par jour ?**

☐	☐	☐	☐	☐	☐	☐	☐
0 jour	1	2	3	4	5	6	7 jours

11 **As-tu déjà fumé du tabac (au moins une cigarette, un cigare ou une pipe) ?**
1 ☐ Oui
2 ☐ Non

12 **Tous les combien fumes-tu du tabac actuellement ?**
1 ☐ Chaque jour
2 ☐ Au moins une fois par semaine, mais pas tous les jours
3 ☐ Moins d'une fois par semaine
4 ☐ Je ne fume pas

13 **Combien as-tu fumé de cigarettes au cours des 30 derniers jours ?**
1 ☐ Aucune
2 ☐ Moins d'une cigarette par semaine
3 ☐ Moins d'une cigarette par jour
4 ☐ 1-5 cigarettes par jour
5 ☐ 6-10 cigarettes par jour
6 ☐ 11-20 cigarettes par jour
7 ☐ Plus de 20 cigarettes par jour

14 **Dans quelle classe étais-tu lorsque tu as fumé ta première cigarette (si tu as fumé ta première cigarette durant les vacances d'été indique la classe dans laquelle tu étais avant les vacances) ?**
0 ☐ Je n'ai jamais fumé de cigarette
Lorsque j'ai fumé ma première cigarette, j'étais en :
1 ☐ CM1
2 ☐ CM2
3 ☐ 6e
4 ☐ 5e
5 ☐ 4e
6 ☐ 3e
7 ☐ 2nde
8 ☐ CAP
9 ☐ Autre classe, précise laquelle : ……

15 **Actuellement, tous les combien bois-tu des boissons alcoolisées, par exemple bière, vin ou alcools forts ?**
◆ *Essaye de compter même les fois où tu n'as bu que de petites quantités.*
◆ *Coche une seule case pour chaque ligne.*

	Jamais	Rarement	Chaque mois	Chaque semaine	Chaque jour
1 Bière (sauf bière sans alcool)	☐	☐	☐	☐	☐
2 Vin	☐	☐	☐	☐	☐
3 Alcools forts (eau de vie, cocktail, whisky, vodka, pastis, digestif…)	☐	☐	☐	☐	☐
4 Prémix (bouteille ou canette contenant un mélange de soda et d'alcool : Smirnoff Ice, Boomerang, Voodoo…)	☐	☐	☐	☐	☐
5 Cidre	☐	☐	☐	☐	☐
6 Champagne	☐	☐	☐	☐	☐
9 Autre boisson contenant de l'alcool. Laquelle : ……	☐	☐	☐	☐	☐

16 **As-tu déjà consommé des boissons alcoolisées au point d'être complètement ivre (soûl, soûle) ?**
1 ☐ Non, jamais
2 ☐ Oui, une fois
3 ☐ Oui, 2 ou 3 fois
4 ☐ Oui, 4 à 10 fois
5 ☐ Oui, plus de 10 fois

17 **Combien de fois as-tu fait les choses suivantes <u>au cours des 30 derniers jours</u> ?**
◆ *Coche une seule case pour chaque ligne.*

	Jamais	1 ou 2 fois	3 à 5 fois	6 à 9 fois	10 à 19 fois	20 à 39 fois	40 fois ou plus
1 Fumer des cigarettes	☐	☐	☐	☐	☐	☐	☐
2 Boire une boisson alcoolisée	☐	☐	☐	☐	☐	☐	☐
3 Être ivre	☐	☐	☐	☐	☐	☐	☐

18 **As-tu déjà consommé du cannabis (chichon, joint, shit, herbe, « H », bédo, marijuana…) ?**
◆ *Coche une seule case pour chaque ligne.*

	Jamais	1 ou 2 fois	3 à 5 fois	6 à 9 fois	10 à 19 fois	20 à 39 fois	40 fois ou plus
1 Au cours de ta vie	☐	☐	☐	☐	☐	☐	☐
2 Au cours des 12 derniers mois	☐	☐	☐	☐	☐	☐	☐
3 Au cours des 30 derniers jours	☐	☐	☐	☐	☐	☐	☐

19 **As-tu déjà consommé une ou plusieurs de ces drogues <u>au cours des 12 derniers mois</u> ?**
◆ *Coche une seule case pour chaque ligne.*

	Jamais	1 ou 2 fois	3 à 5 fois	6 à 9 fois	10 à 19 fois	20 à 39 fois	40 fois ou plus
1 Ecstasy	☐	☐	☐	☐	☐	☐	☐
2 Amphétamines, speed	☐	☐	☐	☐	☐	☐	☐
3 Héroïne	☐	☐	☐	☐	☐	☐	☐
4 Médicaments pour te droguer	☐	☐	☐	☐	☐	☐	☐
5 Cocaïne	☐	☐	☐	☐	☐	☐	☐
6 Colle ou solvants respirés	☐	☐	☐	☐	☐	☐	☐
7 Mop	☐	☐	☐	☐	☐	☐	☐
8 LSD ou acide	☐	☐	☐	☐	☐	☐	☐
9 Produits dopants pour le sport	☐	☐	☐	☐	☐	☐	☐
10 Champignons hallucinogènes	☐	☐	☐	☐	☐	☐	☐
11 Autre drogue. Laquelle : ……	☐	☐	☐	☐	☐	☐	☐

20 **À quel âge as-tu fait les choses suivantes pour la première fois ?**
◆ *S'il y a quelque chose que tu n'as pas fait, coche « jamais ».*
◆ *Coche une seule case pour chaque ligne.*

	Jamais	11 ans ou moins	12 ans	13 ans	14 ans	15 ans	16 ans ou plus
1 Boire une boisson alcoolisée (plus qu'un petit peu)	☐	☐	☐	☐	☐	☐	☐
2 Être ivre après avoir bu une boisson alcoolisée	☐	☐	☐	☐	☐	☐	☐
3 Fumer une cigarette (plus qu'une bouffée)	☐	☐	☐	☐	☐	☐	☐
4 Fumer du cannabis (joint, « H » shit, herbe, chichon, bédo...)	☐	☐	☐	☐	☐	☐	☐

21 **Voici quelques phrases à propos de ton collège ou ton lycée. Indique à quel point tu es d'accord ou non avec chacune d'entre elles.**
◆ *Coche une seule case pour chaque ligne.*

	Tout à fait d'accord	D'accord	Ni d'accord ni pas d'accord	Pas d'accord	Pas du tout d'accord
1 Les élèves de ma classe ont du plaisir à être ensemble.	☐	☐	☐	☐	☐
2 La plupart des élèves de ma classe sont gentils et prêts à aider les autres.	☐	☐	☐	☐	☐
3 Les autres élèves m'acceptent comme je suis.	☐	☐	☐	☐	☐
4 Si j'ai des problèmes au collège ou au lycée, mes parents sont prêts à m'aider.	☐	☐	☐	☐	☐
5 Si besoin, mes parents sont prêts à venir au collège ou au lycée, parler avec mes professeurs.	☐	☐	☐	☐	☐
6 Mes parents m'encouragent à bien travailler en classe.	☐	☐	☐	☐	☐
7 Mes parents m'aident à faire mes devoirs et apprendre mes leçons à la maison, si j'en ai besoin.	☐	☐	☐	☐	☐
8 Je trouve le travail scolaire difficile.	☐	☐	☐	☐	☐
9 Je trouve le travail scolaire fatiguant.	☐	☐	☐	☐	☐

22 **Selon toi, comment tes professeurs trouvent tes résultats scolaires comparés à ceux de tes camarades ?**
1 ☐ Très bons
2 ☐ Bons
3 ☐ Moyens
4 ☐ En dessous de la moyenne

23 **Actuellement, que penses-tu du collège ou du lycée ?**
1 ☐ Je l'aime beaucoup
2 ☐ Je l'aime un peu
3 ☐ Je ne l'aime pas beaucoup
4 ☐ Je ne l'aime pas du tout

24 **Es-tu stressé(e) par le travail scolaire ?**
1 ☐ Pas du tout
2 ☐ Un peu
3 ☐ Assez
4 ☐ Beaucoup

25 **En moyenne, quels étaient tes résultats scolaires à la fin du trimestre dernier (ta moyenne générale) ?**
1 ☐ 16 à 20 sur 20
2 ☐ 14 à 15 sur 20
3 ☐ 12 à 13 sur 20
4 ☐ 10 à 11 sur 20
5 ☐ 8 à 9 sur 20
6 ☐ 6 à 7 sur 20
7 ☐ Moins de 6 sur 20
8 ☐ Je ne sais pas

26 **Au cours de ta vie, as-tu déjà redoublé une classe ?**
0 ☐ Non
1 ☐ Oui, une fois, j'ai redoublé le/la :
2 ☐ Oui, deux fois ou plus, j'ai redoublé les classes suivantes :

Ta santé

27 **Dirais-tu que ta santé est :**
1 ☐ excellente
2 ☐ bonne
3 ☐ assez bonne
4 ☐ mauvaise

28 **Combien pèses-tu (sans vêtements) ?**
.... kilos

29 **Combien mesures-tu (sans chaussures) ?**
1, ... mètre

30 **Voici le dessin d'une échelle. Au sommet de l'échelle, « 10 » est la meilleure vie possible pour toi, tout en bas, « 0 » est la pire vie possible pour toi. En général, où dirais-tu que tu te trouves sur l'échelle en ce moment ?**
◆ *Coche la case en face du nombre qui décrit au mieux où tu te trouves.*
10 ☐ Meilleure vie possible
9 ☐
8 ☐
7 ☐
6 ☐
5 ☐
4 ☐
3 ☐
2 ☐
1 ☐
0 ☐ Pire vie possible

31 **Durant les 6 derniers mois, tous les combien as-tu eu ?**
◆ *Coche une seule case pour chaque ligne.*

	À peu près chaque jour	Plus d'une fois par semaine	Environ une fois par semaine	Environ une fois par mois	Rarement ou jamais
1 Mal à la tête	☐	☐	☐	☐	☐
2 Mal au ventre	☐	☐	☐	☐	☐
3 Mal au dos	☐	☐	☐	☐	☐
4 Des difficultés à t'endormir	☐	☐	☐	☐	☐
5 Des étourdissements	☐	☐	☐	☐	☐

32 **Durant les 6 derniers mois, tous les combien as-tu été ?**
◆ *Coche une seule case pour chaque ligne.*

	A peu près chaque jour	Plus d'une fois par semaine	Environ une fois par semaine	Environ une fois par mois	Rarement ou jamais
1 Déprimé(e)	☐	☐	☐	☐	☐
2 Irritable ou de mauvaise humeur	☐	☐	☐	☐	☐
3 Nerveux (se)	☐	☐	☐	☐	☐

33 **Le mois dernier, as-tu pris des médicaments pour soigner les troubles suivants ?**
◆ *Coche une seule case pour chaque ligne.*

	Non	Oui, une fois	Oui, plus d'une fois
1 Mal à la tête	☐	☐	☐
2 Mal au ventre	☐	☐	☐
3 Difficultés à t'endormir	☐	☐	☐
4 Nervosité	☐	☐	☐
5 Autre chose, précise :	☐	☐	☐

34 **Es-tu porteur d'une maladie chronique ou d'un handicap (comme diabète, allergie ou infirmité motrice cérébrale) ayant été diagnostiqué par un médecin ?**
1 ☐ Non
2 ☐ Oui
Si tu as une maladie chronique ou handicap, écris son nom : ......

35 **Prends-tu des médicaments pour ta maladie chronique ou ton handicap ?**
1 ☐ Je n'ai pas de maladie chronique ni de handicap
2 ☐ Oui
3 ☐ Non

36 **Est-ce que ta maladie chronique ou ton handicap gêne ta présence et ta participation à l'école ?**
1 ☐ Je n'ai pas de maladie chronique ni de handicap
2 ☐ Oui
3 ☐ Non

37 **Penses-tu que ton corps est :**
1 ☐ beaucoup trop maigre
2 ☐ un peu trop maigre
3 ☐ à peu près au bon poids
4 ☐ un peu trop gros
5 ☐ beaucoup trop gros

◆ *Cette question (38) ne concerne que les filles.*

38 **As-tu déjà eu tes règles ?**
1 ☐ Non, je ne suis pas encore réglée
2 ☐ Oui, j'ai commencé à avoir mes règles à l'âge de : .... ans et......... mois

◆ *Pour les garçons et les filles*

39 **As-tu déjà eu un(e) petit(e) ami(e) ?**
1 ☐ Oui
2 ☐ Non

40 **As-tu déjà embrassé ton (ta) petit(e) ami(e) sur la bouche ?**
1 ☐ Oui
2 ☐ Non

41 **As-tu déjà eu des rapports sexuels (on dit aussi « fait l'amour ») ?**
1 ☐ Oui
2 ☐ Non

42 **Quel âge avais-tu quand tu as eu des rapports sexuels pour la première fois ?**
0 ☐ Je n'ai jamais eu de rapports sexuels
1 ☐ 11 ans ou moins
2 ☐ 12 ans
3 ☐ 13 ans
4 ☐ 14 ans
5 ☐ 15 ans
6 ☐ 16 ans
7 ☐ 17 ans ou plus

43 **Ton partenaire pour cette première fois était...**
0 ☐ Je n'ai jamais eu de rapports sexuels
1 ☐ De sexe masculin
2 ☐ De sexe féminin

44 **De ce premier rapport sexuel, tu dirais...**
0 ☐ Je n'ai jamais eu de rapports sexuels
1 ☐ Je voulais qu'il ait lieu à ce moment (ou plus tôt)
2 ☐ J'aurais préféré qu'il ait lieu plus tard
3 ☐ Je ne me suis pas posé la question
4 ☐ Je n'avais pas envie d'avoir ce rapport

45 **Lors de ton dernier rapport sexuel, quelle(s) méthode(s) as-tu (ou ton/ta partenaire) utilisée(s) pour éviter une grossesse ?**
0 ☐ Je n'ai jamais eu de rapports sexuels
1 ☐ Aucune méthode n'a été utilisée pour éviter une grossesse → passe à la question 46

		Oui	Non
2	Pilule contraceptive	☐	☐
3	Préservatifs	☐	☐
4	Retrait	☐	☐
5	Pilule du lendemain	☐	☐
6	Une autre méthode	☐	☐
7	Je ne suis pas sûr(e)	☐	☐

46 **Lors de ton dernier rapport sexuel, as-tu (ou ton/ta partenaire) utilisé un préservatif ?**
0 ☐ Je n'ai jamais eu de rapports sexuels
1 ☐ Oui
2 ☐ Non

Brimades

On dit qu'un(e) élève EST BRIMÉ(E) lorsqu'un(e) autre élève ou un groupe d'élèves lui disent ou lui font des choses méchantes ou qui ne lui plaisent pas. On parle aussi de brimade quand on se moque de manière répétée d'un(e) élève d'une façon qui ne lui plaît pas, ou quand on le (la) met délibérément de côté. Par contre, si deux élèves de la même force se disputent ou se battent, on ne peut pas dire que l'un d'eux (l'une d'elles) est brimé(e). De même, on ne parle pas de brimade quand on plaisante pour s'amuser et de manière amicale.

47 **Tous les combien as-tu été brimé(e) à l'école ces 2 derniers mois ?**
1 ☐ Je n'ai pas été brimé(e) à l'école ces 2 derniers mois
2 ☐ Ce n'est arrivé qu'une ou deux fois
3 ☐ 2 ou 3 fois par mois
4 ☐ Environ une fois par semaine
5 ☐ Plusieurs fois par semaine

48 **Tous les combien as-tu participé à brimer un(e) ou des élèves à l'école ces 2 derniers mois ?**
1 ☐ Je n'ai pas participé à brimer un(e) ou des élèves à l'école ces 2 derniers mois
2 ☐ Ce n'est arrivé qu'une ou deux fois
3 ☐ 2 ou 3 fois par mois
4 ☐ Environ une fois par semaine
5 ☐ Plusieurs fois par semaine

49 **As-tu déjà été victime de violence à l'intérieur de l'école ?**
◆ *Attention : tu peux cocher plusieurs cases.*
1 ☐ Non, jamais
2 ☐ Oui, j'ai été frappé(e)
3 ☐ Oui, on m'a volé mes affaires
4 ☐ Oui, j'ai été victime de racket
5 ☐ Oui, d'une autre manière

50 **As-tu peur de la violence à l'école ou aux alentours ?**
◆ *Attention : tu peux cocher plusieurs cases.*
1 ☐ Non, pas vraiment
2 ☐ Oui, j'ai peur d'être frappé(e)
3 ☐ Oui, j'ai peur qu'on me vole mes affaires
4 ☐ Oui, j'ai peur du racket
5 ☐ Oui, d'une autre sorte de violence

51 **Dans les 12 derniers mois, combien de fois as-tu participé à une bagarre ?**
1 ☐ Je n'ai pas participé à une bagarre dans les 12 derniers mois
2 ☐ 1 fois
3 ☐ 2 fois
4 ☐ 3 fois
5 ☐ 4 fois ou plus

Blessures

Beaucoup de jeunes se font mal ou se blessent en faisant des activités comme du sport ou en se bagarrant dans différents endroits comme la rue ou la maison. On parle également de blessures en cas d'intoxications ou de brûlures. Mais les maladies comme la grippe ou la rougeole ne sont pas des blessures.
La question suivante porte sur des blessures que tu aurais pu avoir dans les 12 derniers mois.

---

52 **Dans les 12 derniers mois, combien de fois as-tu été blessé(e) au point de devoir être soigné(e) par une infirmière ou un médecin ?**
1 ☐ Je n'ai pas été blessé(e) dans les 12 derniers mois
2 ☐ 1 fois
3 ☐ 2 fois
4 ☐ 3 fois
5 ☐ 4 fois ou plus

Ta famille

Toutes les familles sont différentes (par exemple, tout le monde ne vit pas avec ses deux parents, certains ne vivent qu'avec un seul parent, d'autres ont deux maisons ou vivent dans deux familles) et nous aimerions avoir des renseignements sur la tienne.

---

53 **Réponds à la question suivante pour la maison où tu vis tout le temps ou la plupart du temps et coche les cases des personnes qui y vivent avec toi.**
◆ *Attention : tu peux cocher plusieurs cases.*

ADULTES
1 ☐ Mère
2 ☐ Père
3 ☐ Belle-mère (partenaire, copine ou amie du père)
4 ☐ Beau-père (partenaire, copain ou ami de la mère)
5 ☐ Grand-mère
6 ☐ Grand-père
7 ☐ Je vis dans une famille de placement ou un foyer
8 ☐ Je vis avec quelqu'un d'autre ou dans un autre endroit. Écris lequel : ……

ENFANTS
Dis le nombre de frères et sœurs qui vivent dans cette maison (compte aussi tes demi-frères ou sœurs, les autres enfants de tes parents, les enfants adoptifs). Écris zéro (0) si tu n'en as pas.
◆ *Ne te compte pas.*
Combien de frères ? ……
Combien de sœurs ? ……

---

54 **As-tu une autre famille ou une autre maison, comme dans le cas de parents divorcés ou séparés ?**
1 ☐ Non, passe à la question 55
2 ☐ Oui : tous les combien y habites-tu ?
  1 ☐ La moitié du temps
  2 ☐ Régulièrement, mais moins de la moitié du temps
  3 ☐ Parfois
  4 ☐ Presque jamais
◆ *Si tu as une autre famille ou une autre maison, coche les cases des personnes qui y vivent avec toi.*
◆ *Attention : tu peux cocher plusieurs cases.*

ADULTES
1 ☐ Mère

2 ☐ Père
3 ☐ Belle-mère (partenaire, copine ou amie du père)
4 ☐ Beau-père (partenaire, copain ou ami de la mère)
5 ☐ Grand-mère
6 ☐ Grand-père
7 ☐ Je vis dans une famille de placement ou un foyer
8 ☐ Je vis avec quelqu'un d'autre ou dans un autre endroit. Écris lequel : ......

ENFANTS
Dis le nombre de frères et sœurs qui vivent dans cette maison (compte aussi tes demi-frères ou sœurs, les autres enfants de tes parents, les enfants adoptifs). Écris zéro (0) si tu n'en as pas.
◆ *Ne te compte pas.*
Combien de frères ? ......
Combien de sœurs ? ......

---

55 **Dans quelle mesure penses-tu que ta famille est financièrement à l'aise ?**
Ma famille est :
1 ☐ très à l'aise
2 ☐ plutôt à l'aise
3 ☐ moyennement à l'aise
4 ☐ très peu à l'aise
5 ☐ pas à l'aise du tout

---

56 **Ton père a-t-il un travail ?**
1 ☐ Oui
2 ☐ Non
3 ☐ Je ne sais pas
4 ☐ Je n'ai plus (pas) de père ou je ne le vois pas

Si ton père a un travail, indique dans quel type d'endroit il travaille (par exemple : hôpital, banque, restaurant...) :
......
Indique exactement le travail que ton père y fait (par exemple : professeur, chauffeur de bus...) :
......
Si NON, pourquoi ton père n'a-t-il pas de travail ?
◆ *Coche la case qui décrit le mieux sa situation.*
1 ☐ Il est malade, retraité ou étudiant
2 ☐ Il cherche du travail
3 ☐ Il s'occupe des autres ou est à la maison à plein temps
4 ☐ Je ne sais pas

56 **Ta mère a-t-elle un travail ?**
1 ☐ Oui
2 ☐ Non
3 ☐ Je ne sais pas
4 ☐ Je n'ai plus de mère ou je ne la vois pas

Si ta mère a un travail, indique dans quel type d'endroit elle travaille (par exemple : hôpital, banque, restaurant...) :
......
Indique exactement le travail que ta mère y fait (par exemple : professeur, chauffeur de bus...) :
......
Si NON, pourquoi ta mère n'a-t-elle pas de travail ?
◆ *Coche la case qui décrit le mieux sa situation.*
1 ☐ Elle est malade, retraitée ou étudiante
2 ☐ Elle cherche du travail
3 ☐ Elle s'occupe des autres ou est à la maison à plein temps
4 ☐ Je ne sais pas

---

57 **Jusqu'à quel point ta mère sait-elle vraiment...**
◆ *Coche une case par ligne.*

	Elle sait beaucoup	Elle sait un peu	Elle ne sait rien du tout	Je n'ai plus de mère ou je ne la vois pas
1 Qui sont tes ami(e)s	☐	☐	☐	☐
2 Comment tu dépenses ton argent	☐	☐	☐	
3 Où tu es après l'école	☐	☐	☐	
4 Où tu vas le soir	☐	☐	☐	
5 Comment tu passes ton temps libre	☐	☐	☐	

58 **Jusqu'à quel point ton père sait-il vraiment…**

◆ *Coche une case par ligne.*

	Il sait beaucoup	Il sait un peu	Il ne sait rien du tout	Je n'ai plus (pas) de père ou je ne le vois pas
1 Qui sont tes ami(e)s	☐	☐	☐	☐
2 Comment tu dépenses ton argent	☐	☐	☐	
3 Où tu es après l'école	☐	☐	☐	
4 Où tu vas le soir	☐	☐	☐	
5 Comment tu passes ton temps libre	☐	☐	☐	

59 **Ma mère…**

◆ *Coche une case par ligne.*

	Presque toujours	Parfois	Jamais	Je n'ai plus de mère ou je ne la vois pas
1 M'aide autant que j'en ai besoin	☐	☐	☐	☐
2 Me laisse faire les choses que j'aime	☐	☐	☐	
3 M'aime, est affectueuse	☐	☐	☐	
4 Comprend mes problèmes et mes soucis	☐	☐	☐	
5 Aime que je prenne moi-même mes décisions	☐	☐	☐	
6 Essaie de contrôler tout ce que je fais	☐	☐	☐	
7 Me traite comme un bébé	☐	☐	☐	
8 Me fait aller mieux quand ça ne va pas	☐	☐	☐	

60 **Mon père…**

◆ *Coche une case par ligne.*

	Presque toujours	Parfois	Jamais	Je n'ai plus (pas) de père ou je ne le vois pas
1 M'aide autant que j'en ai besoin	☐	☐	☐	☐
2 Me laisse faire les choses que j'aime	☐	☐	☐	
3 M'aime, est affectueux	☐	☐	☐	
4 Comprend mes problèmes et mes soucis	☐	☐	☐	
5 Aime que je prenne moi-même mes décisions	☐	☐	☐	
6 Essaie de contrôler tout ce que je fais	☐	☐	☐	
7 Me traite comme un bébé	☐	☐	☐	
8 Me fait aller mieux quand ça ne va pas	☐	☐	☐	

61 **Que fait ta mère quand tu fais quelque chose qu'elle pense être mal ?**
◆ *Coche une case pour chaque ligne.*

	Très souvent	Souvent	Parfois	Rarement	Jamais	Je n'ai plus de mère ou je ne la vois pas
1 Ma mère ne me punit pas, elle ne le remarque même pas	☐	☐	☐	☐	☐	☐
2 Ma mère m'explique ce que j'ai fait de mal et pourquoi je suis puni(e)	☐	☐	☐	☐	☐	
3 Ma mère me dit que j'ai mal agi, mais elle ne me punit pas	☐	☐	☐	☐	☐	
4 Ma mère me punit immédiatement sans me dire pourquoi	☐	☐	☐	☐	☐	

62 **Que fait ton père quand tu fais quelque chose qu'il pense être mal ?**
◆ *Coche une case pour chaque ligne.*

	Très souvent	Souvent	Parfois	Rarement	Jamais	Je n'ai plus (pas) de père ou je ne le vois pas
1 Mon père ne me punit pas, il ne le remarque même pas	☐	☐	☐	☐	☐	☐
2 Mon père m'explique ce que j'ai fait de mal et pourquoi je suis puni(e)	☐	☐	☐	☐	☐	
3 Mon père me dit que j'ai mal agi, mais il ne me punit pas	☐	☐	☐	☐	☐	
4 Mon père me punit immédiatement sans me dire pourquoi	☐	☐	☐	☐	☐	

63 **Est-il facile ou non pour toi de parler des choses qui te préoccupent vraiment (des choses importantes, graves…) avec les personnes suivantes ?**
◆ *Coche une seule case pour chaque ligne.*

	Très facile	Facile	Difficile	Très difficile	Je n'ai plus (pas) ou je ne vois pas cette personne
1 Père	☐	☐	☐	☐	☐
2 Beau-père (partenaire, copain ou ami de la mère)	☐	☐	☐	☐	☐
3 Mère	☐	☐	☐	☐	☐
4 Belle-mère (partenaire, copine ou amie du père)	☐	☐	☐	☐	☐
5 Frère(s) aîné(s)	☐	☐	☐	☐	☐
6 Sœur(s) aînée(s)	☐	☐	☐	☐	☐
7 Meilleur(e) ami(e)	☐	☐	☐	☐	☐
8 Ami(e) s du même sexe	☐	☐	☐	☐	☐
9 Ami(e) s du sexe opposé	☐	☐	☐	☐	☐

64 **À l'heure actuelle, combien de vrais amis et vraies amies as-tu ?**
◆ *Coche une case pour chaque colonne.*

AMIS	AMIES
1 ☐ Aucun	*1* ☐ Aucune
2 ☐ Un	*2* ☐ Une
3 ☐ Deux	*3* ☐ Deux
4 ☐ Trois ou plus	*4* ☐ Trois ou plus

65 **Généralement, combien de jours par semaine passes-tu du temps avec tes ami(e)s juste après l'école ?**

☐ 0 jour ☐ 1 ☐ 2 ☐ 3 ☐ 4 ☐ 5 ☐ 6 jours

66 **Généralement, combien de soirs par semaine sors-tu avec tes ami(e)s ?**

☐ 0 jour ☐ 1 ☐ 2 ☐ 3 ☐ 4 ☐ 5 ☐ 6 ☐ 7 jours

67 **Combien de jours par semaine passes-tu du temps avec tes ami(e)s à parler au téléphone, à leur envoyer des messages (textos…) ou à les contacter par Internet ?**
1 ☐ Rarement ou jamais
2 ☐ 1 ou 2 jours par semaine
3 ☐ 3 ou 4 jours par semaine
4 ☐ 5 ou 6 jours par semaine
5 ☐ Chaque jour

68 **Combien d'heures par jour environ regardes-tu habituellement la télévision (y compris des films vidéo et des DVD) pendant ton temps libre ?**
◆ *Coche une case pour les jours de semaine et une pour le week-end*

SEMAINE (sauf le mercredi)	WEEK-END
1 ☐ Aucune	*1* ☐ Aucune
2 ☐ Environ une demi-heure par jour	*2* ☐ Environ une demi-heure par jour
3 ☐ Environ 1 heure par jour	*3* ☐ Environ 1 heure par jour
4 ☐ Environ 2 heures par jour	*4* ☐ Environ 2 heures par jour
5 ☐ Environ 3 heures par jour	*5* ☐ Environ 3 heures par jour
6 ☐ Environ 4 heures par jour	*6* ☐ Environ 4 heures par jour
7 ☐ Environ 5 heures par jour	*7* ☐ Environ 5 heures par jour
8 ☐ Environ 6 heures par jour	*8* ☐ Environ 6 heures par jour
9 ☐ Environ 7 heures ou plus par jour	*9* ☐ Environ 7 heures ou plus par jour

69 **Combien d'heures par jour environ joues-tu habituellement à des jeux sur un ordinateur ou sur une console (Playstation, Xbox, Gamecube, etc.) pendant ton temps libre ?**
◆ *Coche une case pour les jours de semaine et une pour le week-end.*

SEMAINE (sauf le mercredi)	WEEK-END
1 ☐ Aucune	*1* ☐ Aucune
2 ☐ Environ une demi-heure par jour	*2* ☐ Environ une demi-heure par jour
3 ☐ Environ 1 heure par jour	*3* ☐ Environ 1 heure par jour
4 ☐ Environ 2 heures par jour	*4* ☐ Environ 2 heures par jour
5 ☐ Environ 3 heures par jour	*5* ☐ Environ 3 heures par jour
6 ☐ Environ 4 heures par jour	*6* ☐ Environ 4 heures par jour
7 ☐ Environ 5 heures par jour	*7* ☐ Environ 5 heures par jour
8 ☐ Environ 6 heures par jour	*8* ☐ Environ 6 heures par jour
9 ☐ Environ 7 heures ou plus par jour	*9* ☐ Environ 7 heures ou plus par jour

70 **Combien d'heures par jour environ utilises-tu habituellement un ordinateur pour participer à des forums de discussion (des « chats »), surfer sur Internet, envoyer du courrier électronique (des e-mails), faire des devoirs pendant ton temps libre ?**
◆ *Coche une case pour les jours de semaine et une pour le week-end.*

SEMAINE (sauf le mercredi)	WEEK-END
1 ☐ Aucune	*1* ☐ Aucune
2 ☐ Environ une demi-heure par jour	*2* ☐ Environ une demi-heure par jour
3 ☐ Environ 1 heure par jour	*3* ☐ Environ 1 heure par jour
4 ☐ Environ 2 heures par jour	*4* ☐ Environ 2 heures par jour
5 ☐ Environ 3 heures par jour	*5* ☐ Environ 3 heures par jour
6 ☐ Environ 4 heures par jour	*6* ☐ Environ 4 heures par jour
7 ☐ Environ 5 heures par jour	*7* ☐ Environ 5 heures par jour
8 ☐ Environ 6 heures par jour	*8* ☐ Environ 6 heures par jour
9 ☐ Environ 7 heures ou plus par jour	*9* ☐ Environ 7 heures ou plus par jour

71 **Combien d'ordinateurs ta famille possède-t-elle ?**
1 ☐ Aucun
2 ☐ Un
3 ☐ Deux
4 ☐ Plus de deux

72 **Est-ce que ta famille a une voiture (ou une camionnette) ?**
1 ☐ Non
2 ☐ Oui, une
3 ☐ Oui, deux ou plus

73 **Est-ce que tu as une chambre pour toi tout(e) seul(e) ?**
1 ☐ Non
2 ☐ Oui

74 **Durant les 12 derniers mois, combien de fois as-tu voyagé avec ta famille pour partir en vacances ?**
1 ☐ Jamais
2 ☐ Une fois
3 ☐ Deux fois
4 ☐ Plus de deux fois

75 **En dehors des heures d'école, combien de FOIS par semaine fais-tu habituellement du sport pendant ton temps libre au point de transpirer ou d'être essoufflé(e) ?**
1 ☐ Chaque jour
2 ☐ 4 à 6 fois par semaine
3 ☐ 2 à 3 fois par semaine
4 ☐ Une fois par semaine
5 ☐ Une fois par mois
6 ☐ Moins d'une fois par mois
7 ☐ Jamais

76 **En dehors des heures d'école, combien d'HEURES par semaine fais-tu habituellement du sport pendant ton temps libre au point de transpirer ou d'être essoufflé(e) ?**
1 ☐ Aucune
2 ☐ Environ une demi-heure
3 ☐ Environ 1 heure
4 ☐ Environ 2 à 3 heures
5 ☐ Environ 4 à 6 heures
6 ☐ 7 heures ou plus

77 **Te sens-tu fatigué(e) en te levant le matin des jours de classe ?**
1 ☐ Rarement ou jamais
2 ☐ Parfois
3 ☐ 1 à 3 fois par semaine
4 ☐ Plus de 4 fois par semaine

78 **D'habitude, combien de temps mets-tu pour t'endormir ?**
◆ *Attention : tu peux cocher plusieurs cases.*
1 ☐ 10 minutes ou moins
2 ☐ 11-20 minutes
3 ☐ 21-30 minutes
4 ☐ 31-40 minutes
5 ☐ Le plus souvent plus de 40 minutes

79 **Quand tu n'as PAS classe le lendemain matin, d'habitude dans ta chambre, le soir, tu utilises… Attention : tu peux cocher plusieurs cases.**
1 ☐ Des livres
2 ☐ Un appareil pour écouter de la musique (lecteur CD, baladeur, lecteur MP3, Ipod)
3 ☐ Une radio
4 ☐ Une télé
5 ☐ Un ordinateur sans Internet
6 ☐ Un ordinateur avec Internet
7 ☐ Une console de jeu (y compris portable)
8 ☐ Un téléphone fixe
9 ☐ Un téléphone portable
10 ☐ Un téléphone portable avec Internet

80 **Quand tu as classe le lendemain matin, d'habitude dans ta chambre, le soir, tu utilises… Attention : tu peux cocher plusieurs cases.**
1 ☐ Des livres
2 ☐ Un appareil pour écouter de la musique (lecteur CD, baladeur, lecteur MP3, Ipod)
3 ☐ Une radio
4 ☐ Une télé
5 ☐ Un ordinateur sans Internet
6 ☐ Un ordinateur avec Internet
7 ☐ Une console de jeu (y compris portable)
8 ☐ Un téléphone fixe
9 ☐ Un téléphone portable
10 ☐ Un téléphone portable avec Internet

81 **Quand tu n'as PAS classe le lendemain matin, d'habitude, à quelle heure t'endors-tu ?**
◆ *Coche une seule case.*
1 ☐ Pas plus tard que 21 h (9 h du soir)
2 ☐ Environ 21 h 30 (9 h et demie du soir)
3 ☐ Environ 22 h (10 h du soir)
4 ☐ Environ 22 h 30 (10 h et demie du soir)
5 ☐ Environ 23 h (11 h du soir)
6 ☐ Environ 23 h 30 (11 h et demie du soir)
7 ☐ Environ minuit
8 ☐ Environ 0 h 30 (minuit et demie)
9 ☐ Environ 1 h (1 h du matin)
10 ☐ Environ 1 h 30 (1 h et demie du matin)
11 ☐ Environ 2 h (2 h du matin)
12 ☐ Environ 2 h 30 (2 h et demie du matin)
13 ☐ 3 h (3 h du matin) ou plus tard

82 **<u>Quand tu as classe</u> le lendemain matin, d'habitude, à quelle heure t'endors-tu ?**
◆ *Coche une seule case.*
1 ☐ <u>Pas plus tard que</u> 21 h (9 h du soir)
2 ☐ Environ 21 h 30 (9 h et demie du soir)
3 ☐ Environ 22 h (10 h du soir)
4 ☐ Environ 22 h 30 (10 h et demie du soir)
5 ☐ Environ 23 h (11 h du soir)
6 ☐ Environ 23 h 30 (11 h et demie du soir)
7 ☐ Environ minuit
8 ☐ Environ 0 h 30 (minuit et demie)
9 ☐ Environ 1 h (1 h du matin)
10 ☐ Environ 1 h 30 (1 h et demie du matin)
11 ☐ Environ 2 h (2 h du matin)
12 ☐ Environ 2 h 30 (2 h et demie du matin)
13 ☐ 3 h (3 h du matin) <u>ou plus tard</u>

83 **<u>Quand tu n'as PAS classe le matin</u>, d'habitude, à quelle heure te réveilles-tu ?**
◆ *Coche une seule case.*
1 ☐ <u>Pas plus tard que</u> 5 h du matin
2 ☐ Environ 5 h 30 du matin
3 ☐ Environ 6 h du matin
4 ☐ Environ 6 h 30 du matin
5 ☐ Environ 7 h du matin
6 ☐ Environ 7 h 30 du matin
7 ☐ Environ 8 h du matin
8 ☐ Environ 8 h 30 du matin
9 ☐ Environ 9 h du matin
10 ☐ Environ 9 h 30 du matin
11 ☐ Environ 10 h du matin
12 ☐ Environ 10 h 30 du matin
13 ☐ Environ 11 h du matin
14 ☐ Environ 11 h 30 du matin
14 ☐ Midi <u>ou plus tard</u>

84 **<u>Quand tu as classe</u> le matin, d'habitude, à quelle heure te réveilles-tu ?**
◆ *Coche une seule case.*
1 ☐ <u>Pas plus tard que</u> 5 h du matin
2 ☐ Environ 5 h 30 du matin
3 ☐ Environ 6 h du matin
4 ☐ Environ 6 h 30 du matin
5 ☐ Environ 7 h du matin
6 ☐ Environ 7 h 30 du matin
7 ☐ Environ 8 h du matin
8 ☐ Environ 8 h 30 du matin
9 ☐ Environ 9 h du matin
10 ☐ Environ 9 h 30 du matin
11 ☐ Environ 10 h du matin
12 ☐ Environ 10 h 30 du matin
13 ☐ Environ 11 h du matin
14 ☐ Environ 11 h 30 du matin
14 ☐ Midi <u>ou plus tard</u>

Si tu le souhaites, tu peux maintenant écrire ce que tu penses de ce questionnaire.
