# Supplementary material for: Changes in tooth brushing frequency and its associated factors from 2006 to 2014 among French adolescents: Results from three repeated cross sectional HBSC studies
Source: PLoS One. 2021 Mar 29;16(3):e0249129. doi: 10.1371/journal.pone.0249129 (PMC8007017; doi:10.1371/journal.pone.0249129)
Supplement: Attachment — Submitted filename: answer reviewerplos.docx [file pone.0249129.s003.docx]

Reviewer #1: The study has important information about the tooth brushing frequency among adolescents. Moreover, the number of included students is good.
Some points were raised and they should be reviewed to improve some parts of the paper.

INTRODUCTION
Please reformulate this phrase to a better understanding: “Based on data from the 2010 French HBSC study (Health Behavior in School-aged Children), a previous work highlighted the strong relationships between tooth brushing frequency and other health behaviors (mostly dietary) on the one hand, and environmental socio-economic factors on the other (16).”

*Answer: The phrase was reformulated as follow: “A previous work on data from the 2010 French HBSC study (Health Behavior in School-aged Children) highlighted the strong relationships between tooth brushing frequency, health behaviors (mostly dietary), health and body perception, and environmental socio-economic factors (16). “*

METHODS
The questionnaire could be provided as supplementary file. It would be easier to the reader to understand what questions were used.

*Answer: All variables used are described in table 1. We also provided many references concerning the protocol. The 2010 French questionnaire has been added as a supplementary file.*

“Despite being fairly constant over the years, the questionnaire has evolved slightly, especially as far as questions specific to the French version are concerned. A shorter questionnaire that does not include the most sensitive items is submitted to the youngest children (11- and 13- year-old groups). Variables coming from items that remained similar in each study from 2006 to 2014, and which were put to all the students, were considered for this paper (Table 2).” If the new version of the questionnaire did not include the sensitive questions, how does this situation interfere in the results of the study? What were these questions? Explain more in the methods and discussion.

*Answer: Those sensitive questions and the resulting variables were not used in this study and were just here as an example of how the questionnaires may be slightly different between children.*

*We reformulated the whole paragraph and deleted the elements that had no impact on this study:*

*“Despite being fairly constant over the years, the questionnaire has evolved slightly, especially as far as questions specific to the French version are concerned. Variables previously identified as associated with toothbrushing frequency and coming from items that remained similar in each study from 2006 to 2014 were considered for this paper (Table 1). They were divided in 4 main categories: Diet, Health and body, School life, Socio-economic status (SES).*

RESULTS
“In particular, healthy eating habits (daily consumption of fruit, vegetables or breakfast) were associated with an increased proportion of twice a day tooth brushing among students (from 67.3% to up to 77.4%).”- this information is in table 2? Table 2 shows the total number or a comparison of the years? Make sure that this information is clear.

*Answer: The previous sentence specify “Among the whole sample (2006, 2010 and 2014 grouped together), almost all studied variables were associated with tooth brushing frequency (Table 1).”*

*We reformulated the sentence: “In particular, healthy eating habits (daily consumption of fruit, vegetables or breakfast) were associated with an increased proportion of twice a day tooth brushing among students; up to a 10% increase for fruit consumption (Table 1).”*

*The table’s legend specifically says “among the whole sample”. We think this is clear enough.*

“In the final regression model, all of the other predictors showed a strong statistical significance with p<0.0001 (while the threshold was only at p<0.001).” – A significance with p<0.0001 does not mean a strong statistical significance. The predictors were statistically significant because were p<0.001. It is possible to say that there is a strong effect analyzing the OR.

*Answer: This sentence no longer exists since the method was changed.*

DISCUSSION
Deeper explanations of the possible reasons of the increase of twice a day tooth brushing should be explored in the first paragraph of the discussion. This part is not clear for me. In the middle of the discussion, it is explained that the program had improved and this can be the reason of this change. So, the first paragraph does not match with the rest of the discussion. It is necessary to clarify it.

*Answer: Discussion was fully rewritten.*

*While this paper’s goal was to study the changes that happened (and not their origin), we discussed potential reasons for the observed changes:*

*“We observed an increase of twice a day tooth brushing among children from around 70% in 2006 and 2010 to 80% in 2014. It is likely that knowledge about the right toothbrushing led to a declaration bias overestimating the real toothbrushing frequency. It is however likely that this bias stayed constant over the 3 studies as no important changes were made to the protocol of the studies, and thus that the increase of twice a day toothbrushing reflect a real trend. Societal pressure may have increased, but would be more likely to be associated with an increase in missing answers to the question in 2014, than to false declaration to this extent. A detailed investigation on the French national and regional prevention programs could help understand this evolution.”*

Reviewer #2: PONE-D-20-01876 Evolution of tooth brushing frequency and its associated factors from 2006 to 2014 among French adolescents

The purpose of this study was to quantify the change from 2006 to 2010 to 2014 in the percentage of adolescents who brushed their teeth twice a day and the association of socio-demographic factors with those changes. Data were drawn from three surveys of French adolescents ages 11, 13, and 15 conducted in 2006, 2010, and 2014 (French cross-sectional studies of the Health Behavior in School-aged Children survey). After exclusions, the study sample was drawn from 7,135 surveys from 2006, 6,103 surveys from 2010, and 5,489 surveys from 2014. Surveys were analyzed using backward stepwise logistic regression with brushing twice a day (y/n) as the outcome. Because I have serious concerns about the use of backward stepwise regression, I will not summarize the results, discussion, or conclusion.

The paper seeks to address an interesting question. It is generally well-written and clear.

I had a few more important concerns, which I include should the authors consider submitting this manuscript to a different journal.

1. I actually wasn’t entirely sure what the research question was. I believe the question was to determine whether the percentage adolescents who brushed their teeth twice a day changed over time. What was confusing was that the predictors were all individual/family level variables. So there seemed to be a mismatch between the question and the factors examined. To determine predictors of change over time, I would look to broader social factors. The authors seemed to recognize this, because in their discussion, they speculated about the role of a social program on the outcome. In their study, however, they did not examine the effect of this social program analytically. Thus, there is a mismatch between the research question and the predictors included in the study. To me, this is a fatal flaw of the paper.

*Answer: It seems our secondary objective was misunderstood by this reviewer which led to many criticisms. We wanted to determine if the relationship between toothbrushing frequency and its predictors changed over time among French adolescents. We did NOT aim to determine predictors of changes in toothbrushing frequency; a goal that would require a longitudinal study. We did NOT aim to offer an analytical model.*

*We reformulated our objectives : “The objectives of the present study were (1) to provide an update on the evolution of the tooth brushing frequency among French adolescents, and (2) to determine if the relationship between toothbrushing frequency and its socio-economic and behavioral predictors changed over the years, using the data from the successive French HBSC studies of 2006, 2010 and 2014.”*

2. The authors seemed to have ideas about the importance of different classes of predictors, but they failed to describe any theoretical underpinning for their ideas. I believe we have passed the time when atheoretical research is acceptable. At this time, the best way for research to make a contribution is if it is theory-driven. The authors should ground their work in theory. Doing so would help the authors address the point above. This is a flaw that is addressable, but addressing it would turn this into a new paper.

*Answer: We indeed have theory about the roles of the identified predictors, which are discussed in the paper. We rewrote the discussion to make it clearer. However, we insist on the fact that this article’s main goal was to identify changes in adolescent toothbrushing, and is thus a descriptive article whose goal is to offer information about the situation, not to offer a full theory on the relationships between toothbrushing and all its associated factors.*

3. The analytic strategy is weak. There are serious drawbacks to using stepwise regression, one being that results using this approach are not generalizable. The results are contingent on the specific sample on which the analysis is run. See: https://www.stata.com/support/faqs/statistics/stepwise-regression-problems/
This is a flaw that is addressable, but addressing it would turn this into a new paper.

My advice would be to start with theory and figure out the ways in which their study can help inform our understanding of that theory.

*Answer: We rewrote the article, deleted the part about stepwise regression and choose to present only descriptive models to make it clearer that this is a descriptive article. We never aimed to offer more than a description of toothbrushing frequency and its associated factors among French adolescents. Our discussion mentions the possible consequences and origin of these associations, but we did not aim to offer a full theory about the mechanisms leading to these associations.*

Minor issues:
1. p. 2, Abstract Objectives, Instead of saying “evolution of tooth brushing frequency,” I might say “trends in the percentage of adolescents who brush their teeth twice a day.”

*Answer: We reformulated to “This study aimed to evaluate in the changes in the percentage of adolescents who brush their teeth twice a day”*

2. p. 2, Abstract Objectives, Instead of saying “… impact of socio-economic status and …,” I might say “association with socio-economic…” “Impact” suggests causality, and I don’t think you can determine causality with these data.

*Answer: We applied this change.*

3. p. 4, Materials and Methods, I am fine with referring the reader to other sources for details, but a few details should be repeated in this paper. I would like to know whether the samples were representative. I assume they were, but I would like that included in this paper explicitly.

*Answer: It is of course a representative sample; we explicitly wrote it. Details were added about the protocol. “We used the data from the representative HBSC cross-sectional studies”.*

4. p. 6, Results, paragraph on the final regression model, I am unclear about the meaning of “… were between the ‘Normal weight’ … and were considered as missing at random.” If the children were non-respondent to the BMI question, how can it be said they were between the two groups? And what does it mean to be between the two groups?

*Answer: We clarified “Among the children with a missing BMI, 67.2% brushed twice a day. Since this value is between the “Normal weight” (72.6% of them brush twice a day) and the “Overweight or obesity” (59.6%) groups, we assumed that those missing value were from children with normal weight as much as overweight or obese children. The most probable reason for those missing value is children not knowing their height or weight. Those missing values were considered as missing at random.”*

5. Table 2. For each variable examined, in addition to including the percentage brushing twice a day, I would also like to know the percentage of the sample endorsing each option. So, for example, for “Alcohol daily,” I would like to know what percentage of the sample endorsed “yes” and what percentage of the sample endorsed “no.”

*Answer: We added this information in the table..*

6. Table 2. The labels for the vast majority of the variables were intuitively obvious, but a few were unclear to me and could use more explanation. What is “priority education,” and what is “Demand from school?”

*Answer: The “priority eduction zone” variables was not studied for this draft. It designed specific places/schools where more money was invested in education due to socio-economic difficulties among the population. “Demand from school” was reformulated as “School too demanding”. In the questionnaire, it refers to school work being too hard and tiring.*

7. Table 2. Because “Demand from school” and “Family structure” were not significant, they should not be bold.

*Answer: We applied this modification.*

I have an additional comment that is not relevant to the research question the authors are examining. The children who were age 11 in 2006 were born in the same year as the children who were 15 in 2010. Similarly, the children who were age 11 in 2010 were born in the same year as the children who were 15 in 2014. If you connect the 11 year olds in 2006 with the 15 year olds in 2010 and connect the 11 year olds in 2010 with the 15 year olds in 2014, you will see that the two resulting slopes differ. Identifying what accounts for the difference in those slopes would be really interesting. I assume it would be something like the social program described in the paper. Remember, though, that lots of other things were changing at the same time. Instagram, for example, also started being used more widely. It could be that increased concern about one’s appearance over the time period accounts for the difference in the slopes. To address this question, I’m not sure whether the difference in differences approach could be used (see: https://en.wikipedia.org/wiki/Difference_in_differences) because the authors don’t have true panel data.
