# Supplementary material for: Changes in tooth brushing frequency and its associated factors from 2006 to 2014 among French adolescents: Results from three repeated cross sectional HBSC studies
Source: PLoS One. 2021 Mar 29;16(3):e0249129. doi: 10.1371/journal.pone.0249129 (PMC8007017; doi:10.1371/journal.pone.0249129)
Supplement: Attachment — Submitted filename: response reviewers.docx [file pone.0249129.s004.docx]

**Answer to the reviewers**

Dear Reviewers,

Thank you for your constructive comments. You will find in this text, in red, all the changes we have made (re-written sentences, corrections, and adjunctions), following your remarks and advices. Changes in the article are in revision mode.

Please find below our answers concerning some of your questions and points:

All comments from reviewers are answered below in the response file. Additionally, here are our answers to the Journal requirements:

1) Concerning style requirements, we updated the organization of the article, the naming of figures and the organization of tables according to PLOS ONE's style requirements. We used page break to keep tables organized since they are quite long.

2) Concerning consent, we corrected the sentence to include more details: "Parents and students were given a consent form with the option to refuse before the adolescent’s participation."

3) Concerning data availability, we realized that the data are now old enough to be on public access, and we gave the link to the databases, both on editorial manager and in the manuscript: "Data are fully available on: https://www.uib.no/en/hbscdata/113290/open-access"

Reviewer #1: Thank you very much for submitting your research to Plos One. The reviewer would like to make several comments on your article.

1.What is the main message the authors would like to deliver?

The main message we would like to deliver is in our conclusion: *“Tooth brushing frequency increased among adolescents from 2010 to 2014, Although socio-economic inequalities in oral hygiene habits persists, populations at risk still benefitted from the improvement in toothbrushing frequency like the rest of the population.Oral hygiene stays closely associated with other healthy behaviors.”*

2.Pleaes comment on the accuracy of the self-reported questionnaire.

We made changes in the discussion about the potential response bias: *“It is likely that knowledge about the right toothbrushing led to a response (social desirability) bias overestimating the real toothbrushing frequency.”*, as well as in the Methods *“The full protocol of the HBSC studies has been described in previous articles and is designed to collect the most representative data and limit the risk of bias (17-19).”.*

Please note that the 3 provided references discuss extensively the accuracy and validity of the questionnaire, taking up to 18 pages in the French book about the 2010 study. Our word count for this article is limited and does not allow us to discuss these matters more extensively.

3.Please provide the full paragraph of self-reported questionnaires.

The 2010 questionnaire has been provided as supplementary data.

4.Please comment on this.
This cutoff was chosen to match the international recommendations for tooth brushing and to avoid groups of subjects being too small.

International recommendations are for a twice a day toothbrushing. We choose to compare children who brushed at least twice a day as advised versus children who did not.

Another element that led us to this choice is the fact that very few children (less than 1%) brushed “never” or “less than once a week”. Unpublished analysis showed us that the low frequency of these answers stayed similar over the years. Previous analysis (Fernandez de Grado G, Ehlinger V, Godeau E, Sentenac M, Arnaud C, Nabet C, et al. Socioeconomic and behavioral determinants of tooth brushing frequency: results from the representative French 2010 HBSC cross-sectional study. J Public Health Dent. 2018;78(3):221-30.) showed us that their characteristics were similar to the “at least once a day but not everyday group”.

5.Please comment on confounding factor.

We made changes to the full paragraph in the discussion which is dedicated to the interpretation of confounding factors:

*“An adequate toothbrushing frequency is the fruit of knowledge (Health literacy) and motivation. Age, gender and SES are most likely predictors of both elements and possibly causal factors for toothbrushing frequency. On the other hand, the “Health and body” and “Dietary behavior” variables are consequences of health literacy and should be considered as confusion confounding factors considering toothbrushing frequency. They are however of interest due to the cumulative effect they may have with toothbrushing frequency in preventing or facilitating oral diseases.*

*Variables describing “School life” are harder to interpret. They may be consequences of SES (a low SES is a predictor of lower academic results and a higher risk of bullying), and thus confusion confounding factors, but could also be causal factors for knowledge (better academic results linked to a better health literacy) and motivation (via better relationships with classmates). Those two interpretations are likely coexisting.”*

6.Can you provide the data for more recent years.

Since this study is repeated every 4 years, only data from 2018 are now available. You can find the main international results which were published in 2020 on the HBSC website http://www.hbsc.org/publications/international/. Raw data older than 3 years old are public, but that’s not the case for the 2018 study. Data from 2018 are available on demand as stated in the article: “***For data access enquiries please contact:*** *The HBSC Data Management Centre is based at the Department of Health Promotion and Development in the University of Bergen, Norway. Professor Oddrun Samdal is the Data Manager of the HBSC study.*
***Email:*** mailto:dmc@hbsc.org
***Web:***  http://www.uib.no/en/hbscdata

Considering the time needed for an extensive analysis, it is not possible for us to modify this article to include the 2018 data.

7.Can the individual be followed up.

No. As stated in the Methods *“Information was collected using an anonymous self-reported questionnaire.* Databases do not contain enough information to identify individuals. The HBSC studies are repeated cross sectional studies and do not constitute a cohort. Each study uses a new randomly selected sample which is different from the other studies and it is thus impossible to follow up individual changes of behavior.

8.What is the rationale for toothbrushing frequency?

We added some new references in the introduction to back up the rationale behind the need for a twice a day toothbrushing. References 7 to 10 discuss the adequate toothbrushing frequency. We lack the space to discuss this rationale as other reviewers asked us to shorten the introduction, especially the part on oral health.

*“There is overall national and international consensus to advise a twice a day frequency of tooth brushing to prevent oral diseases (7, 9, 10)”*

9.Please provide the possible explanation.

I assume you are talking about an explanation for the changes we observed. We added hypotheses in the discussion:

*“It is likely that knowledge about the right toothbrushing led to a response (social desirability) bias overestimating the real toothbrushing frequency. It is however likely that this bias stayed constant over the 3 studies as no important changes were made to the protocol of the studies, and thus that the increase of twice a day toothbrushing reflect a real trend. Societal pressure may have increased, but would be more likely to be associated with an increase in missing answers to the question in 2014, than to false declaration to this extent. The national voluntary prevention program mentioned in introduction was updated to be more efficient, however it still only reaches one third of the targeted children and likely can’t be the only cause of the changes we observed. A detailed investigation on the French national and regional prevention programs as well as societal changes could help understand this evolution.”*

10. Please provide the clinical relevance.

I think this sentence in our conclusion address this point:

*“Oral hygiene stays closely associated with other healthy behaviors and should be considered as part of a whole when addressing health education. It is possible to improve oral hygiene and while the whole population could benefit from it, some populations are more in need and could use oral hygiene and diet improvement to reduce their risk for oral diseases.”*

Thank you very much.

Thank you for your help and constructive comments.

Reviewer #2: The paper shows that among French adolescents, toothbrushing frequency changed from 2006 to 2014 using the data of Health Behavior in School-aged Children (HBSC) survey.
This is an interesting study. However, I would like to make some points regarding the manuscript. The article needs to be revised. First, the authors should follow the STROBE guideline if this study is an observational study. Second, the logic is unclear.

TITLE
1) Please add the study design following the STROBE guideline if it is a cross-sectional study.

The title has been changed to: *“Changes in tooth brushing frequency and its associated factors from 2006 to 2014 among French adolescents: results from three repeated cross sectional HBSC studies” in accordance with STROBE guidelines.*

INTRODUCTION
1) The logic is unclear. If this is a descriptive epidemiology, the authors should need hypothesis formulation. The structure of this paper should be changed dramatically. If it’s a cross-sectional study, they need to add hypothesis before aim. The PECO model should be considered.

We reformulated clearly our main hypothesis before the objectives *“Our main hypothesis was that adolescent’s behavior regarding toothbrushing frequency may have improved.”*

We reformulated clearly according to PECO our secondary hypothesis: *“Our secondary question was “Among adolescents, what is the effect of associated factors (Diet, Health and body perception, School life, SES) in 2006, compared to 2010 and 2014 on toothbrushing frequency”*

2) The topic of dental caries occupies most of introduction section. The authors should make it short.

We deleted a part of the introduction about dental caries among French children.

MATERIALS & METHODS
1) Please add some comments following the STROBE checklist.

We better followed the STROBE checklist and joined a checklist indicating where to find each element. Main changes:

*“Briefly, the HBSC studies are cross sectional studies, repeated every 4 years, describing adolescent’s well-being, health behaviors and their social context.”*

*“The full protocol of the HBSC studies has been described in previous articles and is designed to collect the most representative data and limit the risk of bias”*

*“Information was collected over 2 months (22th April to 22th June in 2010) using an anonymous self-reported questionnaire […] It was validated on around 100 adolescents prior to the study, and double checked for translation from English.* *The minimal sample size (around 4500 for each study) was determined by the international HBSC protocol. Missing answers for this question led to exclusion from the analysis.”*

*“Toothbrushing frequency was our main outcome; other variables were considered as potential predictors.”*

2) Children or adolescents? Please unify the expression.

We unified to the term “adolescents” when writing about this study. “Children” is still used if needed when writing about other studies with broader or younger populations in introduction and discussion.

3) The authors stated that “Details on the survey can be found at http://www.hbsc.org.” and “The full protocol of the HBSC studies has been described in previous articles (17-19).” However, the authors should add more details for readers following the STROBE checklist. Furthermore, I could not find any supplemental files.

We added the full STROBE checklist as supplementary file and made appropriate changes.

Please refer to our answer to remark 1) for detailed changes.

We also added the 2010 French questionnaire as supplementary file.

4) If the authors would like to compare toothbrushing frequency in 2010 and 2014 against 2006, and to investigate whether a temporal association between toothbrushing frequency and its related factors changed over time, the statistical analyses should be changed. The logistic regression models should be revised based on the hypothesis/PECO model.

We used a chi square test to compare toothbrushing frequency between years (Figure 3). The year of study is present in each regression model, with the 2006 year as reference (Tables 2). We fail to understand how different analyses/method could better describe this evolution.

Considering related factors, we tried to introduce interaction factors between related factors and year of study in each model, which yielded no significant result, as stated in the article *“The year 2014 was the second strongest associated factor and no interaction between the year of the study and any other variable predicting tooth brushing frequency was found. All predictors stayed almost constant over the years.”.* Unpublished results, using a single regression model for each study, confirmed the fact that the predictor’s Odds Ratio did not change significantly; with the notable exception of age among girls, as stated in the article: *“The diminution over the studies of the impact of age on girls toothbrushing frequency, identified in bivariate analysis, was not strong enough to be kept in the regression model.”*

We feel that this presentation of our statistical analysis manages best to highlight both the evolution of toothbrushing frequency over the years, as well as the impact of each related factor.

RESULTS
1) Please add the characteristic table following the STROBE guideline.

Figure 2 details sex and age characteristics. Figure 3 details toothbrushing frequency. Values are in the text pages 6 and 7.

Table 1 details all other characteristics of the sample in round brackets in the first column.

2) Please the results based on new methods.

As stated, we think that these analyses are the best way to describe our data.

DISCUSSION
1) The data will be changed by new results.

As stated, we think that these analyses are the best way to describe our data.

2) The authors cannot use “predictive” and “improve” because this is a cross-sectional study and not cohort study.

We replaced the two occurrences of “predictive factors” by “predictors”, which are used according to the common statistical meaning: “Predictor variable is the name given to an independent variable used in regression analyses. The predictor variable provides information on an associated dependent variable regarding a particular outcome.” https://methods.sagepub.com/reference/encyc-of-research-design/n329.xml

As such, predictor is equivalent to “associated variable with a significant effect on the outcome”. Of course, this term does not imply causality which can only be determined by a cohort study as you stated.

We only used “improve” when writing about the changes in toothbrushing frequency between 2006/2010 and 2014. We believe that positive evolution of a variable, observed between two subsequent studies, can be described as an improvement.

Note that we never used “improve” to qualify the link between a predictor and toothbrushing frequency, which would imply causality and would be incorrect.

3) What is the sentence, “An adequate toothbrushing frequency is the fruit of knowledge (Health literacy) and motivation”? Please revise it appropriately.

We reformulated and added a reference; *“An adequate toothbrushing frequency comes from the combination of knowledge (Health literacy) and motivation (24).”*

4) In a paragraph, there is only one sentence, “All those results match previous results (16).” The authors should revise the part.

The sentence was moved to a more appropriate place at the end of the paragraph describing the results; and reworked: *“Our findings match previous results (16).”*

5) Please add some comments about limitations, such as a cross-sectional study, no data of important confounders, no generalizability, bias, etc.

A paragraph was added about limitations and possible future studies:

*“Cohort studies are needed to confirm the causality hypothesis we developed in discussion, which cross-sectional studies like HBSC can’t. While this sample is highly representative of the French population, it would be interesting to check if the associations identified during this study are similar in other countries, which could be done using the result from other HBSC studies.”*

6) Please revise the conclusions because this is a cross-sectional study.

We fail to see which elements of the conclusion are inappropriate for repeated cross-sectional studies.

Reviewer #3: - it is based on questionnaire data (survey) that may over or under estimate the real tooth brush frequency.

This is mentioned in discussion: *“It is likely that knowledge about the appropriate toothbrushing frequency led to a response (social desirability) bias overestimating the real toothbrushing frequency.”*

- If the authors presume the incresced toothbrushing frequency in 2014 is related to the educational program, I recommend to mention the subjective evidence uch as statistical analysis.

This is only a discussion element which would need a dedicated study to be evaluated. We reworked the paragraph to make it clearer: *“The national voluntary prevention program mentioned in introduction was updated to be more efficient on the same period, however it still only reaches one third of the targeted children and likely can’t be the only cause of the changes we observed. A detailed investigation on the French national and regional prevention programs as well as societal changes could help understand this evolution.”*

 Thank you for helping us make a better article.
