# Supplementary material for: Changes in tooth brushing frequency and its associated factors from 2006 to 2014 among French adolescents: Results from three repeated cross sectional HBSC studies
Source: PLoS One. 2021 Mar 29;16(3):e0249129. doi: 10.1371/journal.pone.0249129 (PMC8007017; doi:10.1371/journal.pone.0249129)
Supplement: Attachment — Submitted filename: response reviewers2.docx [file pone.0249129.s005.docx]

**Answer to the reviewers**

Dear Reviewers,

Thank you for your constructive comments. You will find in this text, in red, all the changes we have made (re-written sentences, corrections, and adjunctions), following your remarks and advices. Changes in the article are in revision mode.

Please find below our answers concerning some of your questions and points:

1) The authors answered;
   ‘Our secondary question was “Among adolescents, what is the effect of associated factors (Diet, Health and body perception, School life, SES) in 2006, compared to 2010 and 2014 on toothbrushing frequency”’.
   However, the hypothesis is inappropriate, because the participants were not same. We can only investigate the association between tooth brushing frequency in 2006 and related factors in 2006 but not 2010/2014.

Of course, in our study we compared the association between TBF in 2006 and its related factors in 2006, with the association of TBF in 2010 and its related factors in 2010 (and the same in 2014). We did not compare related factors of 2010 or 2014 with TBF in 2006 which would make no sense.

We rewrote the sentence to make this clearer.

*“Among adolescents, what is the effect of associated factors (Diet, Health and body perception, School life, SES) on toothbrushing frequency in 2006, compared to this association in 2010 and 2014 “*

Actually, when studying a specific age group (children from 11 to 15 in our study), it is impossible to keep the same sample over years. However, using successive representative samples, it is possible to compare those samples and then describe an evolution over the years.

2) The title of table 2 is unclear. Please add more detail comments. What is the odds ratio for? Furthermore, if the dependent value is “Tooth brushing at least twice a day” vs. “Tooth brushing once a day or less” in 2014, the independent values should be the data in 2014 but not 2006/2010. The analyses are inappropriate, even though “all predictors stayed almost constant over the years”. The authors can’t separate the table 2 such as 2a, 2b and 2c, too.

We added more details to this table’s title which was unclear. The new title better explains the analyses that were done, without the need to refer to the manuscript’s text.

***“Tables 2: Results from the three different logistic regressions on the whole sample with the adjusted odd-ratios of brushing at least twice a day:*** *The reference level is a boy of 11 y/o in 2006. For example, on table 2a, being a boy of 11 y/o in 2014 gives an aOR of 1.86 in favor of brushing at least twice a day. No significant interactions were identified, so the aOR of brushing at least twice a day for a 15 y/o girl in 2014 versus a 15 y/o girl in 2006 is not significantly different from 1.86.****”***

3) What is the mechanism? Do the authors mean that eating breakfast daily, eating fruits daily, eating vegetables daily, wealth perceived, BMI, excellent perceived health, body image, having academic delay, perceived school grades, classmate’s support, being bullied, bullying others is associated with TBF? Are these factors risk for decreasing TBF? If yes, they should show the appropriate references in the introduction and discuss the mechanisms. If no, the logistic regression analyses should be deleted or revised.

We discuss those elements in discussion, stating that most of the elements associated with toothbrushing frequency are probably confounding factors sharing common causal factors (SES, health knowledge, lifestyle) with toothbrushing frequency. This is however of interest since some of these factors are also predictors of oral health, just like TBF. Of course, those are only hypothesis in discussion since we have no way to determine causality links.

*“A low tooth brushing frequency is associated with poor perceived health, unhealthy eating habits and overweight or obesity, all of these elements being likely consequences of unhealthy lifestyles and poor health knowledge. This should be a major concern since the association of these unfavorable factors leads to an increased risk of oral and general health diseases. Difficulties in school life and low perceived family wealth are often associated with low SES, which is often found as a socio-environmental predictor for poor oral hygiene.* *Our findings match previous results (17).*

*An adequate toothbrushing frequency comes from the combination of knowledge (Health literacy) and motivation (25). Age, gender and SES are most likely predictors of both elements and possibly causal factors for toothbrushing frequency. On the other hand, the “Health and body” and “Dietary behavior” variables are consequences of health literacy and should be considered as confounding factors considering toothbrushing frequency. They are however of interest due to the cumulative effect they may have with toothbrushing frequency in preventing or facilitating oral diseases.*

*Variables describing “School life” are harder to interpret. They may be consequences of SES (a low SES is a predictor of lower academic results and a higher risk of bullying), and thus confounding factors, but could also be causal factors for knowledge (better academic results linked to a better health literacy) and motivation (via better relationships with classmates). Those two interpretations are likely coexisting.”*

4) The authors can’t use the words, “predictors” and “predictive factors”, because this is a cross-sectional study but not a cohort study. If the new results are similar after re-analyses, they may change the conclusion; “Among French adolescents, TBF improved from 2006 to 2014. The TBF was significantly associated with some health behaviors in each year.”

This term still seems confusing despite our explanation in our previous answer that we used “predictor” with the statistical meaning of “associated factor” with no causality implication. Therefore, and to make it clearer to the readers, we replaced every instance of *“predictor”* and *“predictive factor”* by *“associated factor”* or *“factor associated with…”.*

Following your suggestion, we changed our conclusion in the abstract to *“Among French adolescents, TBF improved from 2006 to 2014. TBF was significantly associated with other health behaviors. These associations stayed* *similar in 2006, 2010 and 2014.”*

We really hope that these answers and corrections will meet your expectations.

Best regards,

The authors.
